# Myocardial Injury in COVID-19 and Its Implications in Short- and Long-Term Outcomes

**DOI:** 10.3389/fcvm.2022.901245

**Published:** 2022-05-26

**Authors:** Andrea Izquierdo-Marquisá, Hector Cubero-Gallego, Álvaro Aparisi, Beatriz Vaquerizo, Núria Ribas-Barquet

**Affiliations:** ^1^Department of Cardiology, Hospital del Mar, Barcelona, Spain; ^2^Department of Medicine, Autonomous University of Barcelona, Barcelona, Spain; ^3^Heart Diseases Biomedical Research Group, IMIM (Hospital del Mar Medical Research Institute), Barcelona, Spain; ^4^Medicine Department, Fabra University, Barcelona, Spain

**Keywords:** SARS CoV-2, infection, COVID-19, inflammation, organ failure, biomarkers, prognosis

## Abstract

COVID-19 caused by severe acute respiratory syndrome coronavirus 2 (SARS-CoV-2) is still a pandemic with high mortality and morbidity rates. Clinical manifestation is widely variable, including asymptomatic or mild respiratory tract illness to severe pneumonia and death. Myocardial injury is a significant pathogenic feature of COVID-19 and it is associated with worse in-hospital outcomes, mainly due to a higher number of hospital readmissions, with over 50% mortality. These findings suggest that myocardial injury would identify COVID-19 patients with higher risk during active infection and mid-term follow-up. Potential contributors responsible for myocardial damage are myocarditis, vasculitis, acute inflammation, type 1 and type 2 myocardial infarction. However, there are few data about cardiac sequelae and its long-term consequences. Thus, the optimal screening tool for residual cardiac sequelae, clinical follow-up, and the benefits of a specific cardiovascular therapy during the convalescent phase remains unknown. This mini-review explores the different mechanisms of myocardial injury related to COVID-19 and its short and long-term implications.

## Introduction

In December 2019, the first cases of pneumonia caused by a new virus called Severe Acute Respiratory Syndrome 2 (SARS-CoV-2) were noted in Wuhan, China. This new infection was named Coronavirus disease 2019 (COVID-19) (1) and it disseminated all over the world, being declared as a global pandemic on March, 2020 by the World Health Organization (WHO). It has overloaded many healthcare systems and has been considered the worst sanitary crisis since the pandemics of Influenza in 1918. Despite substantial progress in clinical research, new viral strains are still a challenge for the healthcare system. Therefore, understanding the potential contributors of hospital readmissions after COVID-19 might improve long-term outcomes (2).

## The SARS-CoV-2 Virus

### SARS-CoV-2 Origin

Human epidemiological data suggest a zoonotic origin of SARS-CoV-2 from a Seafood Market in China. Early reports suggested that bats were the most likely initial hosts and its transmission to human involved an intermediate animal Once most of the animal trading markets in China were closed, infected human have become the main source of the infection (3–5).

### SARS-CoV-2 Structure

SARS-CoV-2 is an enveloped ribonucleic acid (RNA) virus with a double-layered lipid envelope. Its name refers to its core shell with surface projections which features a solar corona (Latin: corona = crown). There are four coronaviruses subfamilies: alpha- and beta- subfamilies, originated from mammals (bats); and gamma- and delta- subfamilies, from pigs and birds. While alpha-coronaviruses cause asymptomatic or mildly symptomatic infection, beta-coronaviruses may cause severe disease (6).

SARS-CoV-2 belongs to the beta-coronaviruses, such as Middle East Respiratory Syndrome (MERS-CoV) and SARS-CoV. SARS-CoV-2 and SARS-CoV share around the 80% of their genome (7).

The most important envelope proteins in SARS-CoV-2 are: Spike (S) protein that mediates the viral entry into the host cell through ACE2 receptor; Membrane (M) and Envelope (E) protein which are responsible for the membrane structure. The nucleocapsid is mainly composed of the N protein (8).

### SARS-CoV-2 Transmission Methods

SARS-CoV-2 predominant route of transmission from person-to-person is through respiratory droplets and contact (3). While its infectivity (R0) is around 2.2–2.7, the R0 for SARS-CoV was 3 and 2–5 for MERS-CoV (9).

Droplet transmission occurs when mucous membranes, such as mouth, nose and eyes, are exposed to infectious respiratory droplets of someone within 1 m who has respiratory symptoms. Indirect transmission can occur through fomites on surfaces in the environment around the infected person (e.g., Stethoscope) (10).

Airborne transmission may occur during procedures that generate aerosols: e.g., endotracheal intubation, nebulized treatments, bronchoscopy, tracheostomy, non-invasive positive-pressure ventilation or cardiopulmonary resuscitation (10, 11). Some evidence suggests a fecal-to-oral transmission, but to date it has not been proven (12).

### Pathogenesis

Extrapolations from knowledge about other similar beta-coronaviruses, like SARS-CoV and MERS-CoV, are used to understand SARS-CoV-2 pathogenesis (8, 13–15).

The entrance of the virus into the host cells is mediated by the union between the Spike protein of SARS-CoV-2 and the angiotensin-converting enzyme 2 (ACE2) and protein priming by the serine protease TMPRSS2. TMPRSS2 transcription is regulated by androgenic hormones which can explain, partially (7) the higher mortality and incidence in men.

Previous studies about SARS-CoV showed that the effectiveness of the virus banding to ACE2 could be an important determinant of the virus transmissibility. Consequently, the increased transmissibility of SARS-CoV-2 may be due to its higher affinity of binding to the ACE2 receptor than SARS-CoV (16).

Viral genome replication and translation is held after the cell entry and RNA has been released into the cytoplasm. When this replication occurs in the epithelial cells of the respiratory tract it causes severe pneumonia or Acute respiratory distress syndrome (ARDS) (17).

**FIGURE 1:**
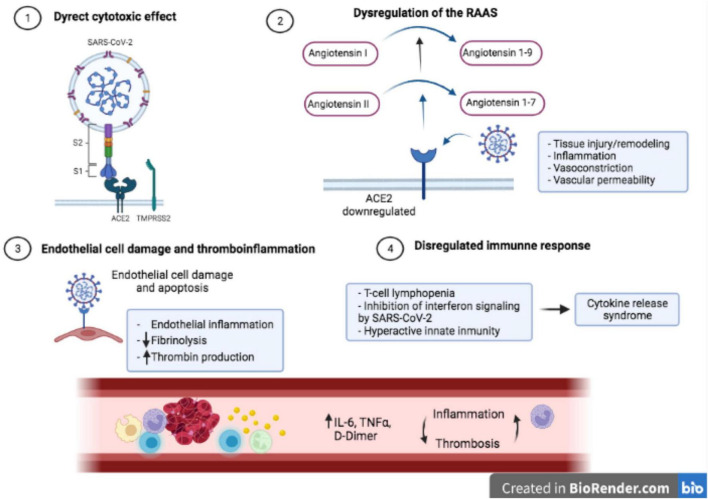
Pathophysiology of COVID-19 [adapted from Gupta et al. (18)]: **(1)** Direct virus-mediated cell damage. SARS-CoV-2 enters into host cells through the union between the spike protein and ACE2 receptor in the presence of TMPRSS2 protease; **(2)** Downregulation of ACE2 leads to a dysregulation of the RAAS and consequently to an increase of angiotensin I and angiotensin II; **(3)** Virus entrance to endothelial cell damage induces apoptosis and endothelialitis; **(4)** T-cell lymphopenia, inhibition of interferon signaling and hyperactive innate immunity produces a dysregulation of the immune response and a cytokine storm syndrome.

Proposed mechanisms for the pathophysiology of multi-systemic injury secondary to SARS-CoV-2 infection are direct cytotoxicity, endothelial cell damage and thrombo-inflammation, dysregulation of the renin-angiotensin–aldosterone system (RAAS) and dysregulation of the immune response (18, 19). The role of each mechanism in the pathophysiology of COVID-19 is still not fully delimited. Some of these mechanisms are unique to COVID-19 (ACE2-mediated viral entry and dysregulation of the RAAS). However, the microcirculation dysfunction and the pathogenesis caused by the systemic release of cytokines are also present in sepsis (20) (Figure 1).

## Myocardial Injury in SARS-CoV-2

The ACE2 receptors are highly expressed in cardiovascular cells and are involved in blood pressure regulation and myocardial function (21). Cardiovascular manifestations of COVID-19 are variable, including myocardial injury, thromboembolism, arrhythmia, acute coronary syndrome, heart failure or cerebrovascular accidents. These cardiovascular complications have been associated with worse short and long-term outcomes (22, 23). The mechanisms of cardiovascular damage are not clearly understood and hypotheses are based on SARS-CoV-2 resemblance to other coronaviruses.

**FIGURE 2:**
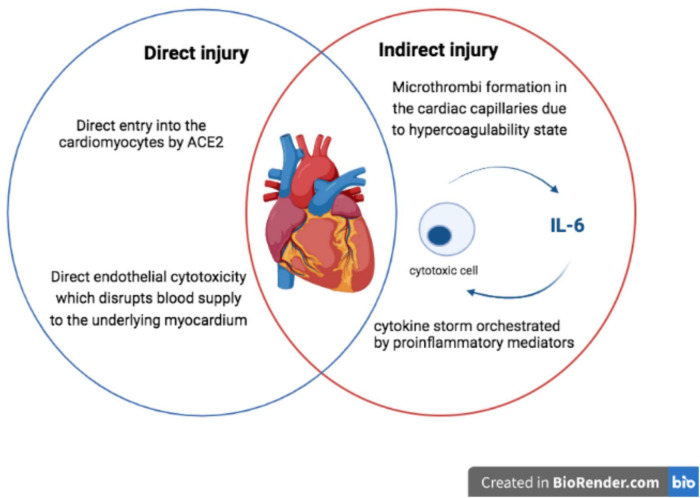
Mechanisms for myocardial damage in COVID-19 [adapted from Siripanthong et al. (72)].

Myocardial injury is diagnosed when serum levels of cardiac troponin (cTn) are above the 99*^th^* percentile upper reference limit (24). Initial studies suggested that myocardial injury was present in around 20–30% of COVID-19 patients (23, 25–29). The incidence of myocardial injury increases with COVID-19 severity and has prognostic implications (30). The suggested mechanisms for SARS-CoV-2-related cardiac damage are: (1) cardiomyocytes injury; (2) endothelial cells injury and endothelialitis; (3) indirect injury from hypercoagulability state; (4) ischemic myocardial injury; and (5) indirect injury from cytokine storm (Figure 2).

### Direct Cardiomyocytes Injury

Myocarditis related to viral infection is widely described (31). Few studies about fulminant myocarditis in COVID-19 patients have been published (32–36) and suggest that direct myocardial infection is produced through the ACE2 receptor. Cardiomyocyte apoptosis induced by SARS-CoV-2 has been proved *in vitro* (37). However, the pathophysiology of this injury is not clearly defined to date, only one study has displayed viral genome particles in the cardiomyocytes (38) while SARS-CoV-2 is principally found inside macrophages or interstitial cells (32, 39, 40).

### Endothelial Cells Injury

Endothelial cells infection by SARS-CoV-2 ends up into cell injury of tissues supplied by the affected vasculature. Fibrin deposition and activation of the terminal portion of the complement cascade in the context of endothelial inflammation has been confirmed in autopsies of COVID-19 patients (41).

### Hypercoagulability State

Thrombotic events such as pulmonary embolism, venous thromboembolism, vascular cerebral accident, and myocardial infarction have been related to COVID-19 disease (42, 43), as well as disseminated intravascular coagulopathy (DIC) in 71% of COVID-19 non-survivors (44). However, the precise mechanisms which activates the coagulation system are not fully understood and are partially attributed to the cytokine storm and the dysregulation of the immune response. In addition to the hypercoagulability and endothelial dysfunction, the immobility of critical patients and the associated venous stasis complete the 3 Virchow criteria for a high risk of venous thrombosis. Finally, COVID-19 treatments would have interactions with antiaggregant and anticoagulant therapies and increase the risk of thromboembolic events (17).

### Myocardial Ischemia

The hypercoagulability and inflammatory stage may lead to myocardial ischemia because of a thrombotic event (type I myocardial infarction) or because of a mismatch between myocardial oxygen supply and demand (type II myocardial infarction). Patients with previous history of cardiovascular disease seem to have a higher risk of myocardial ischemia during viral infections than those without cardiovascular disease (45, 46).

### Cytokine Storm Syndrome

SARS-CoV-2 infection has been related to a cytokine storm that may end up to a systemic inflammatory reaction, sepsis, and multiorgan failure (47). Few studies have suggested myocardial injury in the setting of systemic inflammation but without cardiomyocytes virus infiltration, implying that in this setting, myocardial injury could be related to the cytokine storm (48). Among all cytokines, interleukin-6 (IL-6) has an important position in COVID-19, not only because of its stimulating effects in cytokine storm, but also because of its cardiovascular effects. Some studies have revealed that IL-6 produces cardiac dysfunction as a consequence of decreasing papillary muscles contractility. In addition, IL-6 has been associated with arrhythmic (49) events and higher levels of myocardial injury biomarkers, as a consequence of its role in atherosclerotic events (50–52), cardiac fibrosis (53), pulmonary hypertension (54) and higher cardiovascular risk (55).

## Prognostic Implications of Myocardial Damage in COVID-19 Patients

Myocardial injury is present in around one-third of hospitalized COVID-19 patients (23, 25–29, 56, 57). Higher cTn levels predict worse outcomes in COVID-19 hospitalized patients, including a higher risk of death and mechanical ventilation (Supplementary Table 1). Consequently, the measurement of troponin levels could be a useful tool to guide patient management during their hospitalization (58, 59).

Myocardial injury in COVID-19 patients has been associated with cardiovascular risk factors such as high blood pressure or diabetes mellitus, with heart failure, ischemic cardiovascular disease and chronic renal disease (26, 29, 60). In terms of laboratory findings, it is associated with lower hemoglobin levels and higher inflammatory markers (26, 29, 56).

Cardiovascular inflammation, microvascular dysfunction, ischemia, and myocardial injury, usually found in COVID-19 patients, are known precursors of cardiac arrhythmias and prolonged QT intervals (61, 62). Sinus tachycardia is the most frequently arrhythmia present in COVID-19, probably related to many causes (hypoperfusion, hypoxia, fever…). New onset or preexisting atrial fibrillation is the second most frequent arrhythmia, being present in 10–14% of hospitalized patients and 22% of critical COVID-19 patients (63–65). Atrial fibrillation and sinus tachycardia are independent predictors of severity, myocardial injury, and worse outcomes of COVID-19 patients (65). Regarding ventricular arrhythmias, Guo et al. reported an incidence of malignant ventricular arrhythmias in 6% of hospitalized patients. These findings are similar to those found during influenza infection (66). Another report form Du et al. found that arrhythmias were registered in a 60% of patients but only two patients died because of a malignant arrhythmia (67). Since the beginning of the pandemic, early reports proposed hydroxychloroquine or azithromycin as effective drugs against SARS-CoV-2, further studies found that cardiac arrest was more frequent in patients who received these drugs (68).

To date, only few studies regarding the cardiovascular long-term consequences after recovery fromCOVID-19 have been published (Supplementary Table 1), suggesting worse long-term outcomes (69–72). In our previous published study of a cohort with 172 patients who survived COVID-19 hospitalization, myocardial injury was associated with poor prognosis, mainly due to a higher number of readmissions (71). In the same direction, Kini et al. (70) found that the risk of death at 30 days was significantly increased in those patients who had myocardial injury during the acute phase. Finally, Xie et al. (69) showed that beyond 1 month after infection, COVID-19 patients have higher risk of a cardiovascular event; consequently, specific cardiovascular follow-up should be included in care pathways of COVID-19 survivors.

Myocarditis and myocardial injury related to SARS-CoV-2 infection can produce functional and morphologic sequelae on the heart, particularly in those with preexisting cardiac disease (73–75). Cardiovascular magnetic resonance (CMR) imaging has been used as a tool to assess cardiac involvement in patients who survived COVID-19. A multicenter study with 148 recovered COVID-19 patients (74) showed that myocardial injury was associated with CMR abnormalities in around 50% of the patients. Three different patterns of injury were observed: non-infarct myocarditis-pattern injury (27%), ischemic pathology (22%), and non-ischemic non-specific scar (5%). In a 6% of the patients, dual pathology (ischemic and non-ischemic patterns) were observed. No global functional ventricular consequences were found. In addition, a German study that included patients which were recently recovered from COVID-19, CMR revealed cardiac abnormalities in 78% of patients, such as decreased left ventricular ejection fraction and higher left ventricle volumes. Endomyocardial biopsy in patients with cardiac involvement found in CMR studies, showed active lymphocytic inflammation (75). CMR studies in recovered COVID-19 patients have found some disorders that could be responsible for future arrhythmias or heart failure. Further investigation of long-term cardiovascular consequences of COVID-19 is required.

## Conclusion and Future Perspectives

The COVID-19 pandemic is still causing significant morbidity and mortality worldwide. Close monitoring of cardiovascular system in patients with COVID-19 may help to identify high- vs. low-risk patients. Patients with COVID-19 infection and previous cardiovascular disease present a poor prognosis and a higher risk of overall mortality. Further investigation regarding the mechanism, manifestations, and prognosis of myocardial injury in COVID-19 patients is required to improve therapies and prognosis.

## Author Contributions

AI-M, HC-G, ÁA, BV, and NR-B contributed to the literature review and manuscript drafting. All authors contributed to the article and approved the submitted version.

## Conflict of Interest

The authors declare that the research was conducted in the absence of any commercial or financial relationships that could be construed as a potential conflict of interest.

## Publisher’s Note

All claims expressed in this article are solely those of the authors and do not necessarily represent those of their affiliated organizations, or those of the publisher, the editors and the reviewers. Any product that may be evaluated in this article, or claim that may be made by its manufacturer, is not guaranteed or endorsed by the publisher.
