# Supplementary material for: Myocardial Injury in COVID-19 and Its Implications in Short- and Long-Term Outcomes
Source: Front Cardiovasc Med. 2022 May 26;9:901245. doi: 10.3389/fcvm.2022.901245 (PMC9204594; doi:10.3389/fcvm.2022.901245)
Supplement: Supplementary file 1 [file Table_1.DOCX]

| **Short-term follow-up** | |
| --- | --- |
| **First author (Ref)** | **Title** |
| Bhatraju et al. (25) | Covid-19 in critically ill patients in the Seattle Region - Case series |
| Shi et al. (26) | Association of cardiac injury with mortality in hospitalized patients with COVID-19 in Wuhan, China |
| Liu et al. (27) | The science underlying COVID-19: Implications for the cardiovascular system |
| Yang et al. (28) | Clinical course and outcomes of critically ill patients with SARS-CoV-2 pneumonia in Wuhan, China: a single-centered, retrospective, observational study |
| Guo et al. (28) | Cardiovascular implications of fatal outcomes of patients with coronavirus disease 2019 (COVID-19) |
| Lala et al. (56) | Prevalence and impact of myocardial injury in patients hospitalized with COVID-19 Infection |
| Bardají et al. (57) | Prognostic implications of myocardial injury in patients with and without COVID-19 infection treated in a university hospital |
| Calvo et al. (58) | Markers of myocardial injury in the prediction of short-term COVID-19 prognosis |
| Arentz et al. (59) | Characteristics and outcomes of 21 critically ill patients with COVID-19 in Washington State |
| Kini et al. (70) | Types of myocardial injury and mid-term outcomes in patients with COVID-19 |
| **Long-term follow-up** | |
| **First author (Ref)** | **Title** |
| Xie et al. (69) | Long-term cardiovascular outcomes of COVID-19 |
| Kini et al. (70) | Types of myocardial injury and mid-term outcomes in patients with COVID-19 |
| Izquierdo et al. (71) | Myocardial injury as a prognostic factor in mid- and long-term follow-up of COVID-19 survivors |
| Siripanthong et al. (72) | The pathogenesis and long-term consequences of COVID-19 cardiac injury state-of-the-art review |

**Supplementary table 1: Summary of studies addressing myocardial damage in COVID-19 patients with short- and long-term follow-up**
